# Dyakonov surface waves in dielectric crystals with negative anisotropy

**DOI:** 10.1515/nanoph-2024-0161

**Published:** 2024-05-09

**Authors:** Dmitry A. Chermoshentsev, Evgeny V. Anikin, Ilia M. Fradkin, Mikhail S. Sidorenko, Aleksandra A. Dudnikova, Aleksandr S. Kalganov, Mikhail F. Limonov, Nikolay A. Gippius, Sergey A. Dyakov

**Affiliations:** 366033 Skolkovo Institute of Science and Technology, Moscow, Russia; 528193 Russian Quantum Center, Skolkovo, Moscow, Russia; Moscow Institute of Physics and Technology, Moscow Region, Russia; 65071 ITMO University, St. Petersburg, Russia; 68635 Ioffe Institute, St. Petersburg, Russia

**Keywords:** Dyakonov surface waves, Fabry–Pérot resonance, perturbation theory, effective medium theory, metamaterial

## Abstract

Since the initial discovery of Dyakonov surface waves at a flat infinite interface of two dielectrics, at least one of which is *positively anisotropic*, extensive research has been conducted towards their theoretical and experimental studies in materials with positive anisotropy. The potential applications of these waves were initially limited due to the stringent conditions for their existence and the requirement for position anisotropy. In our study, we present the theoretical prediction and experimental observation of a novel type of Dyakonov surface waves that propagate along the flat strip of the interface between two dielectrics with *negative anisotropy*. We demonstrate that the conditions for surface waves are satisfied for negatively anisotropic dielectrics owing to the specific boundaries of the strip waveguide confined between two metallic plates. We study such modes theoretically by using the perturbation theory in the approximation of weak anisotropy and demonstrate that the electromagnetic field distribution in these modes is chiral. Experimental verification of theoretical predictions is made in the microwave range using 3D-printed negatively anisotropic water-dielectric metamaterial slabs. The existence of Dyakonov surface waves in negative crystals prompts a reassessment of the list of materials suitable for practical realization of these waves in the visible and infrared ranges. Due to the ability of the considered modes to transmit chiral light, they have potential in the sensing of chiral organic molecules.

## 1 Introduction

Dyakonov surface waves (DSWs) are an electromagnetic modes that exist at the interface between two dissimilar materials, at least one of which is anisotropic. In contrast to surface plasmon polaritons, DSWs have no theoretical limit in propagation length as they can exist at the interface of two lossless dielectrics. In Refs. [1], [2], [3] by F. N. Marchevskii, M. I. Dyakonov and N. S. Averkiev in the 80s, it is detailed that DSWs can propagate within a certain angular range relative to the optical axis, and only in systems composed of positively anisotropic materials. Positive optical anisotropy means that *ɛ*_‖_ > *ɛ*_⊥_, where *ɛ*_‖_ (or *ɛ*_⊥_) is the principal value of dielectric permittivity tensor parallel to (or perpendicular to) the optical axis. Since then, extensive research has been performed toward the theoretical studies of Dyakonov-like surface waves at interfaces of different combinations of isotropic, uniaxial, biaxial, and chiral materials with positive anisotropy [4], [5], [6], [7], [8], [9], [10], [11], [12], [13], [14], [15], [16], [17], [18], [19], [20], [21], [22], [23], [24], [25], [26]. A narrow range of propagation angles and the requirement of positive anisotropy sufficiently decrease the number of materials suitable for the practical realization of DSWs. Nevertheless, O. Takayama et al. experimentally observed such waves for the interface formed by a dielectric liquid and biaxial KTP crystal [27]. Later, the same group experimentally demonstrated the existence of Dyakonov-like guided modes in thin aluminum oxide nanosheets placed between anisotropic crystal and dielectric liquid [28].

The existence of DSWs has also been theoretically predicted for interfaces between isotropic materials and metamaterials with artificially designed anisotropy [29], [30], [31], [32], [33], [34], [35], [36], [37], [38]. In such structures, the angular existence domain of DSWs can be significantly extended due to the development of the structure with the required dielectric permittivity tensor. The experimental realization of such DSWs has been reported in Ref. [39].

Very recently, it was shown that Dyakonov-like surface waves could exist as bound states at the interface of cylindrical metamaterials [40]. Subsequently, a significant case of Dyakonov-like surface waves in finite-size resonator structures was studied for cylindrical waveguides [41], as well as for flat interfacial strip waveguides [42], Fabry–Pérot cavities, and rectangular confined planar resonators [43]. It was demonstrated that due to the non-zero curvature of cylindrical waveguides, Dyakonov surface waveguide modes within them inevitably possess radiative losses. Conversely, in flat interfacial strip waveguides, such modes can propagate without radiative losses, akin to classical DSWs.

The unique properties of Dyakonov waves have garnered significant attention from researchers owing to their potential applications in various fields. Dyakonov surface waves can serve as biosensors, detecting biological molecules and entities with high sensitivity [27]. They also offer promise for long-range optical communications [11], imaging, and applications in topological insulators. Demonstrating strong confinement and localization, they offer great opportunities for developing new functionalities in nanophotonic devices [28].

Since the pioneering works [2], [3] demonstrated that the main requirement for materials to support DSWs at their infinite interfaces is being positively anisotropic, the DSWs community has focused on positive anisotropic materials. Until now, there has been no literature data on DSWs in negative anisotropic materials. In this work, we will demonstrate that the interface between two negative anisotropic materials can support DSWs when confined between two metallic plates. To describe this type of DSWs, we develop a theoretical approach based on perturbation theory and the approximation of weak anisotropy. As a result, we theoretically determine the dispersion of the new-type DSWs, calculated their field distribution profiles, demonstrated that these modes are chiral, and finally, estimate their quality factors and propagation losses. To experimentally verify theoretical predictions, we fabricate a negatively-anisotropic water-based metamaterial waveguide [44] and observe surface waves in it in the RF range.

## 2 Theoretical prediction of Dyakonov surface waves in negative crystals

**Figure 1:**
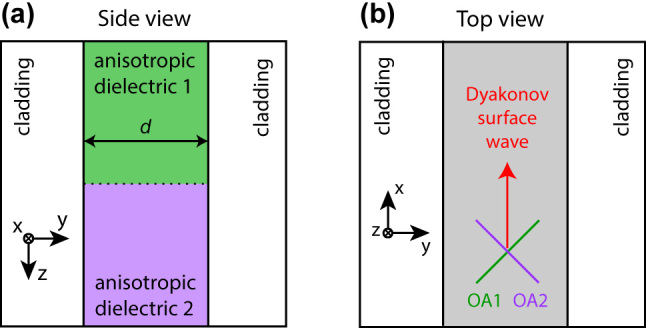
The waveguide for surface waves. (a) Side view and (b) top view of the Dyakonov waveguide. Optical axes (OA) of anisotropic dielectrics 1 and 2 are perpendicular to each other and form the angle of 45° to the waveguide boundaries.

The structure under consideration consists of two spliced dielectric slabs confined by thick metallic plates at |*y*| = *d*/2 (Figure 1(a) and (b)). The slabs are infinite in *x*-direction and semi-infinite in *z*-direction; the interface between them is at *z* = 0. The principal values of the permittivity tensor of both slabs are *ɛ*_*e*_, *ɛ*_*o*_, *ɛ*_*o*_ with *ɛ*_*e*_ < *ɛ*_*o*_, i.e., we consider the case of a negative anisotropy. The values of *ɛ*_*e*_, *ɛ*_*o*_ are assumed to be real and positive. The optical axes of the upper and lower slabs are rotated by an angle of ± 45° about the *z*-axis as shown in Figure 1(b).

We represent the dielectric tensor $\hat{\varepsilon }\left(z\right)$ inside the waveguide as $\hat{\varepsilon }\left(z\right)={\hat{\varepsilon }}_{0}+\delta \hat{\varepsilon }\left(z\right)$ , where

(1) $$\begin{array}{c} {\hat{\varepsilon }}_{0}=\mathrm{diag}\left(\varepsilon_{1},\varepsilon_{1},\varepsilon_{2}\right),\\ \delta \hat{\varepsilon }\left(z\right)=\mathrm{sign}\left(z\right)\left(\begin{array}{ccc} 0 & \delta \varepsilon & 0\\ \delta \varepsilon & 0 & 0\\ 0 & 0 & 0 \end{array}\right), \end{array}$$

with $\varepsilon_{1}=\frac{\varepsilon_{o}+\varepsilon_{e}}{2},\;\varepsilon_{2}=\varepsilon_{o}$ and *δɛ* = *ɛ*_2_ − *ɛ*_1_. In addition to this model, we use two important approximations to develop a theory for DSWs in negative anisotropic crystals. First, we assume that materials have low anisotropy, *δɛ* ≪*ɛ*_1,2_ (the most often case for natural anisotropic crystals), which allows us to consider the Dyakonov waveguide as a small perturbation of a homogeneous anisotropic waveguide with a dielectric tensor ${\hat{\varepsilon }}_{0}$ . Second, we replace metallic plates with perfect electric conductor (PEC) plates, meaning that the metallic cladding can be accounted for simply using specific PEC boundary conditions. For the sake of generality, at the end of this section, we will show that DSWs exist in a more general case when these two approximations are not realized.

The waveguide modes of unperturbed anisotropic waveguide with the PEC walls can be analytically described for arbitrary *k*_*x*_ and *k*_*z*_ as shown in Supplemental Materials. The dispersion curves of the lowest TM and TE modes at *k*_*z*_ = 0 are set by the following equations:

(2) $$\begin{array}{c} {\left(\omega_{k_{x},0}^{TE}\right)}^{2}=\frac{c^{2}}{\varepsilon_{2}}\left[{\left(\frac{\pi }{d}\right)}^{2}+k_{x}^{2}\right],\;\;\;\;\;\;\;\;{\left(\omega_{k_{x},0}^{TM}\right)}^{2}=\frac{c^{2}k_{x}^{2}}{\varepsilon_{1}} \end{array}.$$

One can see from Eq. (2) that these modes intersect, and the wavevector of intersection is the following:

(3) $$k_{x}=\frac{\pi }{d}\sqrt{\frac{\varepsilon_{1}}{\varepsilon_{2}-\varepsilon_{1}}}.$$

It is important to note that the intersection of the lowest TE and TM modes of the unperturbed uniaxial waveguide with *ɛ*_1_ < *ɛ*_2_ occurs only due to specific PEC boundary conditions. We will show that close to the intersection, the perturbation $\delta \hat{\varepsilon }$ leads to considerable mixing of TE and TM modes giving rise to a special type of surface mode. Starting now, we call this electromagnetic mode as Dyakonov surface wave of the II type (DSW-II), while a classical Dyakonov surface wave is referred to as Dyakonov surface wave of the I type (DSW-I). DSW-II does not exist at the infinite interface of two negatively anisotropic plates or at the strip interface of two spliced negatively anisotropic slabs confined between air plates.

In the approximation of a low anisotropy, the intersection of the lowest TM and TE modes’ dispersion curves is far from the next-order waveguide modes (see Supplemental Materials). It enables us to expand the field of DSW-II over the lowest TE and TM modes of the unperturbed waveguide:

(4) $$\begin{array}{l} \vec{E}\left(y,z\right)=\int \frac{dk_{z}}{2\pi }\left[\alpha \left(k_{z}\right){\vec{E}}_{k_{x},k_{z}}^{TE}\left(y\right)\right.\\ \left.+\beta \left(k_{z}\right){\vec{E}}_{k_{x},k_{z}}^{TM}\left(y\right)\right]e^{ik_{z}z}, \end{array}$$

where *α*(*z*) and *β*(*z*) are the slowly varying envelopes and ${\vec{E}}_{k_{x},k_{z}}^{TE}$ and ${\vec{E}}_{k_{x},k_{z}}^{TM}$ are the normalized lowest TE and TM modes, which are described as follows:

(5) $$\begin{array}{c} {\vec{E}}_{k_{x},k_{z}}^{TE}\left(y\right)=\sqrt{\frac{2}{d}}\frac{1}{\varepsilon_{2}^{3/2}\varepsilon_{1}}\frac{c^{2}}{\omega^{2}}\left(\begin{array}{c} \varepsilon_{2}k_{x}k_{z}\cos\frac{\pi }{d}y\\ i\varepsilon_{2}\frac{\pi }{d}k_{z}\sin\frac{\pi }{d}y\\ -\varepsilon_{1}\left[k_{x}^{2}+{\left(\frac{\pi }{d}\right)}^{2}\right]\cos\frac{\pi }{d}y \end{array}\right),\\ {\vec{E}}_{k_{x},k_{z}}^{TM}\left(y\right)=\frac{1}{\varepsilon_{1}\sqrt{d}}\frac{c}{\omega }\left(\begin{array}{c} 0\\ k_{x}\\ 0 \end{array}\right). \end{array}$$

Below we present the result of applying the perturbation theory for finding the expressions of the amplitudes *α*(*z*) and *β*(*z*) and the dispersion for DSW-II (see Supplemental Materials for details on perturbation theory). After plenty of algebraic manipulations, the following formulae can be obtained for the slowly varying envelopes [42]:

(6) $$\begin{array}{c} \alpha \left(z\right)=\frac{\omega^{2}\sigma \delta \varepsilon {\text{e}}^{-\kappa_{2}|z|}}{\left(\gamma_{k_{x},0}^{TE}-\frac{\kappa_{2}^{2}}{2m_{1}}\right)}-\frac{\omega^{2}\sigma \delta \varepsilon {\text{e}}^{-\kappa_{1}|z|}}{\left(\gamma_{k_{x},0}^{TE}-\frac{\kappa_{1}^{2}}{2m_{1}}\right)},\\ \beta \left(z\right)=-i\left(\frac{{\text{e}}^{-\kappa_{2}|z|}}{\kappa_{2}}-\frac{{\text{e}}^{-\kappa_{1}|z|}}{\kappa_{1}}\right), \end{array}$$

where constants *κ*_1,2_ have positive real part and are found as two roots of the characteristic equation

(7) $$\left(\gamma_{k_{x},0}^{TE}-\frac{\kappa^{2}}{2m_{1}}\right)\left(\gamma_{k_{x},0}^{TM}-\frac{\kappa^{2}}{2m_{2}}\right)+\omega^{4}\sigma^{2}\delta \varepsilon^{2}\kappa^{2}=0,$$

where

(8) $$\begin{array}{c} \sigma =\frac{2\sqrt{2\varepsilon_{2}}}{\varepsilon_{1}^{3/2}\pi }\frac{k_{x}}{k_{x}^{2}+{\left(\frac{\pi }{d}\right)}^{2}},\;\;\;\;\;\;\;\;m_{1}^{-1}=m_{2}^{-1}=\frac{2c^{2}}{\varepsilon_{1}} \end{array}.$$

In Eq. (7) we use the notation $\gamma_{k_{x},k_{z}}^{TE\left(TM\right)}={\left(\omega_{k_{x}k_{z}}^{TE\left(TM\right)}\right)}^{2}-\omega^{2}$ for brevity. Please note the quantities 1/*κ*_1_ and 1/*κ*_2_ have a meaning of the DSW-II penetration depths. As it follows from Eq. (7), the smaller the anisotropy constant, the larger the penetration depths; and in the limit a zero anisotropy, the frequency of DSW-II tends to the frequencies of the waveguide modes and the DSW-II becomes completely delocalized.

**Figure 2:**
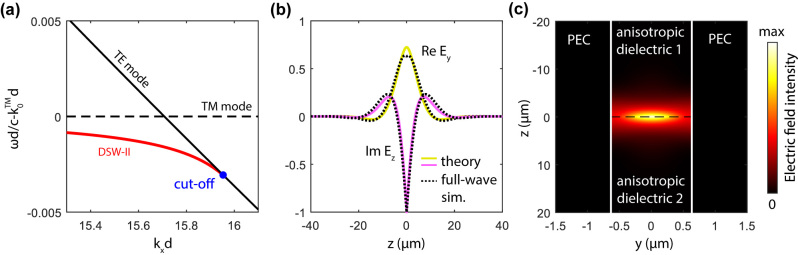
The modal structure of the interfacial waveguide. (a) The dispersions of the DSW-II (red line) and the TE and TM waveguide modes of an anisotropic waveguide (black solid and dashed lines) shown as a difference with the dispersion of the TM waveguide mode. (b) Theoretically calculated fields *E*_*y*_(0, *z*) and *E*_*z*_(0, *z*) (yellow and magenta lines) shown together with COMSOL simulation results (black dashed lines). (c) Theoretically calculated electric field intensity in DSW-II. Please note that in panel (c) the limits of *y*- and *z*-axes are different. Calculations in (b) and (c) are made for *λ* = 1550 nm, *k*_*x*_ *d* = 15.705 and *d* = 1265 nm. Dielectric permittivities for all panels are *ɛ*_*o*_ = 9.75, *ɛ*_*e*_ = 9.0, *δɛ* = 0.375.

The dispersion equation of the surface mode can be obtained from the boundary conditions and have the following form:

(9) $$\sqrt{\gamma_{k_{x},0}^{TE}\gamma_{k_{x},0}^{TM}}=2m{\left(\omega^{2}\sigma \delta \varepsilon \right)}^{2}-\gamma_{k_{x},0}^{TM}.$$

here we use the notation *m* = *m*_1_ = *m*_2_. It turns out that the transcendental Eq. (9) has a solution (red line in Figure 2(a)) and, hence, the DSW-II exists. As expected, the DSW-II dispersion curve is close to the intersection of the lowest TE and TM modes. One can see from Figure 2(a) that there is an upper cut-off for the DSW-II, which is because the solution of Eq. (9) exists when the right-hand side is non-negative.

The DSW-II transforms into the TE mode at the cut-off point and delocalizes completely. The existence of the upper cut-off point of the dispersion curve leads to the fact that there is an upper cut-off waveguide thickness *d* at which the DSW-II ceases to exist. After substitution of the coefficients (8) into the right hand side of Eq. (9) we obtain that the cut-off thickness depends on the frequency *ω* or vacuum wavelength *λ* as follows:

(10) $$d\leq \frac{c\pi }{\omega }\sqrt{\frac{\varepsilon_{1}}{\varepsilon_{2}\delta \varepsilon }}=\frac{\lambda }{2}\sqrt{\frac{\varepsilon_{1}}{\varepsilon_{2}\delta \varepsilon }}.$$

**Figure 3:**
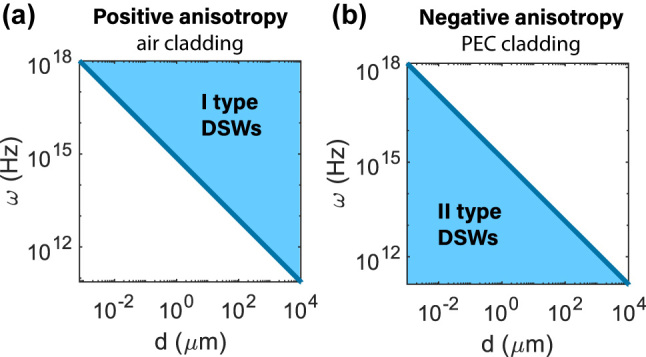
Domains of the existence of (a) the I type and (b) the II type DSWs in the structure shown in Figure 1 with dielectrics with positive or negative anisotropy. In (a) *ɛ*_*o*_ = 9.0, *ɛ*_*e*_ = 9.75, in (b) *ɛ*_*o*_ = 9.75, *ɛ*_*e*_ = 9.0.

In Ref. [42], where a DSW-I in positively anisotropic materials between two air plates are considered, the situation is the opposite. Namely, there is a lower cut-off point for the dispersion curve. Figure 3 visually illustrates the domains of existence of DSW-I and DSW-II in terms of the waveguide thicknesses *d* for different frequencies *ω*. Since for DSW-II, there is the upper cut-off waveguide thickness, DSW-II does not exist in the case of an infinite (*d* → ∞) interface between two anisotropic materials, in contrast to DSW-I.

The distribution of electric field intensity of DSW-II within the waveguide’s vertical cross-section calculated by the formula (4) is shown in Figure 2(c). One can see that the field decays away from the interface and has the maximum in the middle of the waveguide. To verify the developed theory, we calculate the *z*-dependence of the electric field by the formula (4) and compare it with the results of full-wave electromagnetic simulations made in COMSOL (Figure 2(b)). We obtain that apart from a generally perfect agreement between two field profiles, and there is a slight discrepancy between two curves near the interface caused by the influence of the higher-order waveguide modes of the unperturbed waveguide (see Supplemental Materials for details).

**Figure 4:**
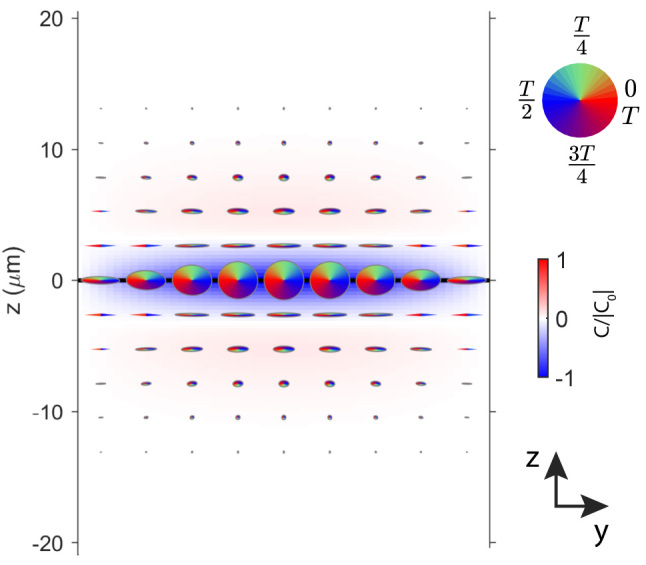
The *yz* cross-section of the electric field distribution in DSW-II mode, calculated for *λ* = 1550 nm and *k*_*x*_ *d* = 15.393. The red-white-blue background represents the spatial function of the local chirality density *C* normalized to the value taken in the middle of the waveguide. The red-blue-green ellipses denote polarization ellipses of the local electric field, with a color corresponding to a certain phase of electromagnetic oscillations (a circular color chart is shown on the right).

Next, an important property of the considered waveguide is its chirality. By definition, an object is chiral if it cannot be superimposed onto its mirror image by rotations or translations. These two mirror-image forms are referred to as enantiomers. By choosing the orientation of the optical axes of the upper and lower anisotropic dielectrics, we select one of the enantiomers of a chiral waveguide. Since chiral optical resonators generally support chiral electromagnetic modes, DSWs at the interface between anisotropic dielectrics should also be chiral. To study the chiral property of DSW-II, we calculate the spatial distribution of local chirality density as [45], [46]

(11) $$C=\frac{\omega }{2c^{2}}Im\left(E\cdot H^{*}\right)$$

and plot this quantity in Figure 4 along with local polarization ellipses of electric field. One can see in Figure 4 that in the vicinity of the interface, the electric field is almost circularly polarized and is chiral. Since chiral molecules interact with left- and right-handed electromagnetic fields differently, DSW-II has a potential to serve as a platform for sensing chiral organic molecules.

## 3 Experimental observation of 2nd type Dyakonov surface waves

The experimental realization of DSW-II waves in natural anisotropic materials poses significant challenges due to two cumbersome problems. The first issue is the generally low anisotropy of natural nonlinear crystals, which leads to a large penetration depth of DSWs. This fact necessitates the fabrication of samples with very specific geometric dimensions, making the task even more challenging, given that the optical axes of anisotropic materials must be oriented at specific angles. The second issue arises from the requirement to implement PEC boundary conditions for the waveguide. In principle, metallic walls can be used for this purpose in the visible and infrared ranges, but material absorption in them will suppress the propagation length of surface waves. Moreover, the metal-dielectric interfaces may support parasitic surface plasmon polaritons, which can hybridize with DSW-II, complicating their experimental observation.

The above problems can be solved by replacing natural anisotropic substances with artificial anisotropic metamaterials. A wide variety of possible metamaterial configurations offer a broad range of effective dielectric permittivities with controllable anisotropy contrast. In this work, a specific realization of this approach is implemented in the microwave range (*λ* > 1 mm), where the fabrication of samples becomes a feasible task, and even thin metal foils can be considered as PEC.

**Figure 5:**
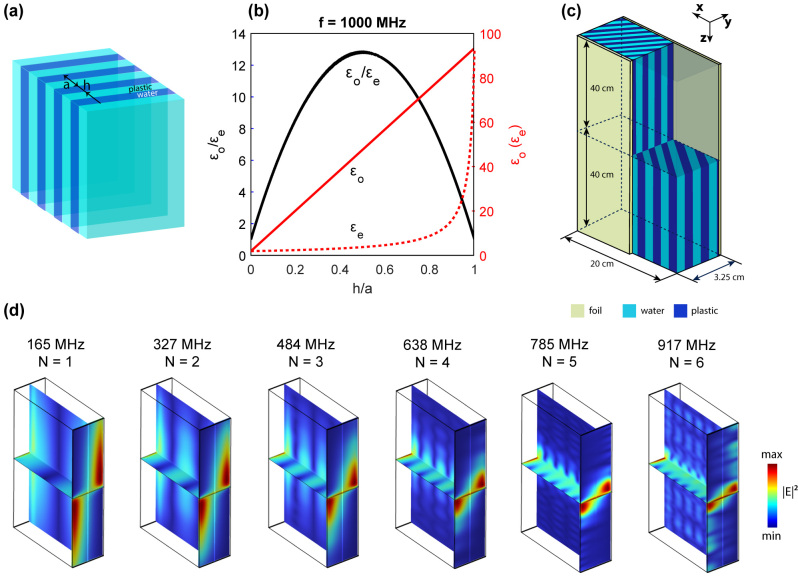
The metamaterial waveguide. (a) 3D scheme of the metamaterial structure; (b) the effective ordinary *ɛ*_*o*_ (red solid) and effective extraordinary *ɛ*_*e*_ (red dashed) dielectric constants as a function of the ratio of the width of polylactide width *h* to the period of structure *a* calculated by effective media theory (see Methods Section 1), the anisotropic contrast dependence *ɛ*_*o*_/*ɛ*_*e*_ is presented by black curve, the simulation was conducted for frequency *f* = 1000 MHz; (c) the schematic view of the experimental sample, different materials are highlighted by colors; (d) electric energy density of Fabry–Pérot-like DSWs-II calculated for *N* = 1.6.

The designed sample consists of two metamaterial slabs of a periodic array of polylactide plastic plates (see Figure 5(a)). This configuration is characterized by negative anisotropy, which, together with the geometrical simplicity of a multi-plates structure, makes this metamaterial an ideal platform for observation of DSW-II in the microwave range. Given the low dielectric permittivity of plastic, the anisotropy contrast of a bare air-plastic metamaterial structure is relatively small. To enhance the anisotropy, we fill the voids between the plastic plates with water. The resulting effective dielectric tensor of such water-based metamaterial [44] is calculated using a current-based homogenization method [47], which is elaborated in the Methods section. As depicted in Figure 5(b), the maximum anisotropy contrast is achieved when the volume filling factors of water and plastic are equal. The optimal configuration, striking a balance between the fabrication feasibility, minimization of scattering losses, experimental frequency, and the validity of the effective medium theory, is achieved with plastic plates’ thickness *h* = 2 mm, metamaterial period of *a* = 4 mm, waveguide width of *W* = 3.25 cm, waveguide length of *L* = 20 cm and waveguide height of *H* = 40 cm. In the designed sample, the plastic plates in the upper and lower slabs are oriented at angles of ± 45° relative to the DSW-II propagation direction, as shown in Figure 5(c). Then, the slabs are covered with aluminum foil on both sides, which ensures PEC boundary conditions in the microwave range used in the experiment.

Although the fabricated sample possesses an interface of two anisotropic materials designed to support DSW-II, its modal structure differs from that of an idealized interfacial waveguide described in Section 2. This difference arises because the fabricated sample has finite sizes in all three dimensions, unlike the theoretical model structure depicted in Figure 1. To be more specific, finite sizes in the *x* and *z*-directions quantize all the modes, imposing an additional condition for the existence of DSW-II:

(12) $$\frac{\pi N}{k_{x}}=L,$$

where *N* is an integer. This condition corresponds to Fabry–Pérot-like DSW-II resonances formed by the superposition of forward and backward propagated DSW-II, as initially proposed in Supplemental Materials of Ref. [43] for DSW-I. The profiles of such a mode, calculated for *N* = 1.6 are shown in Figure 5(d), where the interference nodes and antinodes are clearly visible. An important distinction of Fabry–Pérot-like DSW-II resonances from their counterparts in an infinite-length waveguide, is the antisymmetric field intensity distribution of the former (see Supplemental Materials Section IV).

**Figure 6:**
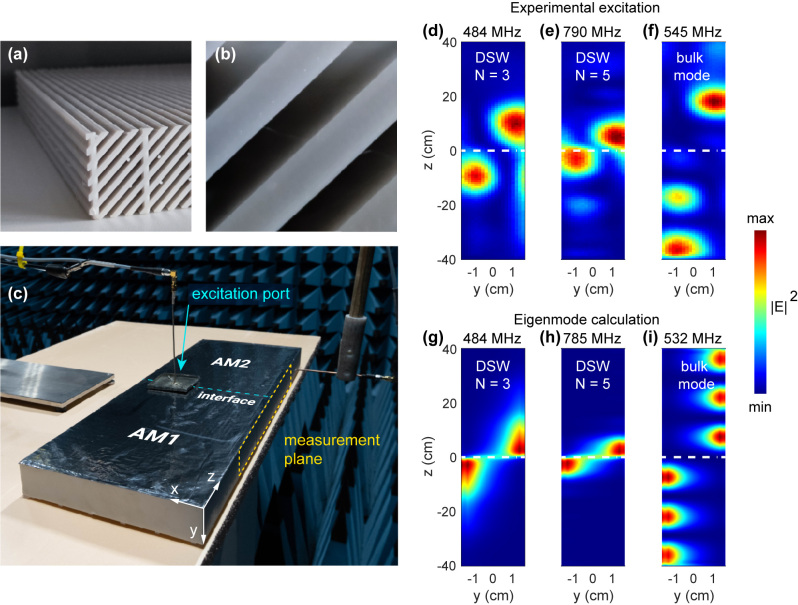
Experimental observation of DSW-II. (a) And (b) fabricated sample of metamaterial slab. (c) Experimental setup. The coaxial input is inserted into the excitation port drilled on the interface, shown by the cyan color. The field distribution measurement is performed on the measurement plane highlighted with yellow. (d)–(f) Experimental excitation field profiles and (g)–(i) theoretical eigenmode field profiles. In panels (d), (e), (g), and (h), the field distributions correspond to DSW-II, while in panels (f) and (i) – to a bulk mode (i.e., Fabry–Perot mode on the entire sample height). The fields are presented by the electric energy density, as shown on the color scale.

Thus, in the fabricated sample (Figure 6(a) and (b)), DSW-II forms a discrete set of eigenmodes, and there is both an upper and lower cut-off frequency for this set. The upper cut-off is discussed in Section 2 and is a characteristic of DSW-II in an infinite-length waveguide. In contrast, the lower cut-off is a feature of DSWs-II confined within a finite-size sample. Note that, at low frequencies, DSW-II are poorly localized (see Figure 5(d) for *N* = 1), and their field profiles are almost indistinguishable from those of the first-order bulk Fabry–Perot modes over the entire height of the structure (2*H* = 80 cm). As a result, in our full-wave simulations in COMSOL, for the given dimensions of the fabricated sample, among other modes, we numerically identify only five eigenmodes localized at the interface. These are the modes which can be achieved experimentally. In Table 1 we present frequencies, Q-factors and figures-of-merit (FOMs) of these modes. The parameter FOM is used for estimation of the propagation distance of Pérot-like DSW-II, and is calculated as

(13) $$FOM=\frac{Re\left(k_{x}\right)}{Im\left(k_{x}\right)}$$

like Ref. [48]. The FOM has a meaning of a DSW-II decay length, expressed in units of the DSW-II wavelength. One can see from Table 1 that the FOM and the Q-factor have opposite behaviours: the FOM decreases with *N* while the Q-factor increases. This can be attributed to the reduced scattering of DSW-II to the bulk Fabry–Pérot modes over the entire height of the structure as a result of the reflection from the boundaries at shorter wavelengths.

**Table 1:** Calculated parameters of Fabry–Pérot-like DSW-II.

*N*	2	3	4	5	6
*ν*, MHz	327	484	638	785	917
*Q*-factor	27.6	37.2	46.3	62.8	106.6
FOM	15,958	4020	2604	1650	1330

To experimentally excite DSW-II in the fabricated sample, we insert a coaxial input near the antinode of the eigenmodes with frequencies of *f* = 484 MHz and 790 MHz and then measure the electric field distribution at the edge of the interfacial waveguide using a network analyzer, as shown in Figure 6(c). The observed electric field distributions at excitation frequencies *f* = 484 MHz and 785 MHz (Figure 6(d) and (e)) are localized in the vicinity of the interface between the upper and lower metamaterials, exhibiting the same asymmetric profile as their numerical counterparts presented in Figure 6(g) and (h). To confirm that this localization is only achieved at specific frequencies defined by Eq. (12), we also measure the electric field distribution at *f* = 545 MHz. We find that shifting the excitation frequency away from the Fabry–Pérot condition for DSW-II results in the delocalization of the field distribution, consistent with numerical simulations, as shown in Figure 6(f), (i).

Although there was a notable consistency between the theoretical and experimental electric field intensity profiles, we observed some disparity in the local polarization distributions between the numerically simulated and experimentally excited waves (see Figure S7 in Supplemental Materials). The reason for such inconsistency is the complexity and non-ideality of the receiving antenna, which introduces phase errors to the results of measurements.

## 4 Discussion

The discovery of DSW-II significantly increases practical interest in Dyakonov surface waves because it is now apparent that they can exist in a much broader range of materials, with feasible excitation conditions, and can propagate in the waveguide regime [41], [42], [43]. More specifically, DSWs can find applications in those areas where field localization near the interface and/or lossless propagation along the waveguide is important.

Among these areas is optical sensing, where due to their surface localization and narrow range of propagation angles, DSWs can potentially allow the development of compact, highly sensitive devices. The strong interaction of DSWs with the surrounding environment enables the detection of refractive index changes due to mechanical deformation of the waveguide or due to the presence of molecules on the surface [49]. This capability can find applications in biosensing, where the detection of biomolecules or pathogens at extremely low concentrations is of utmost importance. By functionalizing the surface with specific receptors, one can harness DSWs for the effective detection and quantification of various biological analytes, providing a highly sensitive and label-free sensing platform.

In sensing applications, one of the most intriguing opportunities provided by DSWs, is their different susceptibilities to chiral organic molecules with different handednesses [50]. Moreover, since electromagnetic field of DSWs at the interface of two anisotropic dielectrics is chiral, eigenfrequency of these modes will depend on relative concentration of left and right molecules. This opens the possibility for DSW-enabled anisotropic interfacial waveguides to become a platform for chiral sensing.

Another perspective for DSWs arises from the fact that many anisotropic materials, both with positive and negative anisotropy, exhibit strong non-linearity in their optical response. This characteristic makes DSW waveguides a useful platform for non-linear photonics [51], [52]. In particular, if the interfacial waveguide is formed by materials exhibiting the Pockels effect (e.g., LiNbO_3_), the presence of metallic walls enables electro-optical frequency tuning of DSWs. This opens the possibility in developing electro-optical modulators on DSWs [53]. However, it is important to note that transitioning from the microwave range to the infrared or optical ranges will cause the electric field to start penetrating the metallic walls, leading to increased absorption and losses within the structure. In this case, the metallic walls can no longer be considered as PECs. Moreover, at high frequencies, the electromagnetic field may excite plasmons that propagate along the metallic walls, which significantly complicates the modal structure of the waveguide. A possible solution to this problem could be to replace the metallic walls with a lossless periodic structure or metamaterial that can effectively be considered as a PEC. Analyzing the properties of DSWs in such structures would require separate research, which is beyond the scope of the current study.

Finally, DSWs can find applications in spintronics, with the main idea of utilizing spin-dependent excitation for information processing and storage [8], [54]. Combination of DSWs and spin currents may lead to developing spintronic devices that operate at high speeds and with low power consumption [55].

From the viewpoint of practical applications, an important advantage of DSWs in waveguide structures over conventional DSWs at infinite interfaces is their robustness with respect to geometrical errors. Unlike conventional DSWs, which face the issue of an extremely narrow range of in-plane propagation angles, DSWs in waveguide structures do not encounter this complication. As soon as the in-plane wavevector of DSW-II is linked to the waveguide width, a small fabrication error in the width of the fabricated waveguide only alters the mode frequency, without negating the mode’s existence. Moreover, DSWs-II appear stable even with non-orthogonal optical axes in the upper and lower anisotropic materials.

To demonstrate this for DSWs-II, we have calculated the dependence of the propagation constant of the surface wave on the angle *α* between the optical axes of two anisotropic dielectrics, ranging from 0° to 180° (see Figure S10 in the Supplemental Materials). We found that at a fixed wavelength (*λ* = 1550 nm), as *α* increases, DSW-II shifts from a TM-to a TE-guided mode, with corresponding change of the field distribution inside waveguide (see Figure S11 in the Supplemental Materials). We also investigated numerically the existence of a Fabry–Pérot-like DSW-II in experimental sample across a wide range of angles *α*, as illustrated in Figure S12. We found that the strongest localization occurs near *α* = 90°, while at extremely large or small *α* values, the mode becomes delocalized. In principle, deviations of *α* from 90° do not reduce the rotational symmetry of the entire structure; therefore, a strict condition of *α* = 90° does not automatically guarantee the strongest localization or the longest propagation length of DSW-II, unlike in the case of conventional DSWs. Further research is needed to explore the impact of the angle *α* on the properties of DSWs in waveguide structures.

## 5 Conclusions

In conclusion, we have conducted a theoretical study of a novel type of Dyakonov surface waves propagating along the flat interface strip between two uniaxial dielectrics with negative anisotropy. We demonstrated that the conditions for these surface waves are met in negatively anisotropic dielectrics, thanks to the specific boundaries of the strip interfacial waveguide confined between two metallic plates. We theoretically analyzed these modes using a perturbative approach under the assumption of weak anisotropy. We have demonstrated that these modes are chiral. We also have presented the experimental observation of the II-type of Dyakonov surface wave in the radio frequency range, within a negatively anisotropic water–dielectric metamaterial sample. The experimentally measured electric field distribution of the quantized DSW-II resonance corresponds to the superposition of forward- and backward-traveling 2nd-type Dyakonov surface waves reflecting from the opposite edges of the waveguide. The observed antisymmetric field distribution aligns with our numerical modeling. Finally, we believe that our findings open up a new, unexplored research area in the field of surface waves and broaden the list of materials suitable for the practical realization of Dyakonov surface waves. This fact significantly increases practical interest in these surface waves, which now become much more feasible for applications in optical communication systems, biosensing, non-linear photonics and spintronics.

## 6 Methods

### 6.1 Derivation of effective dielectric tensor of anisotropic metamaterial

The effective permittivity tensor of the considered metamaterial structure can be easily derived via the current-based homogenization procedure proposed in [47]. This approach proved itself to be reliable and easy to realize. In particular, we apply COMSOL Multiphysics computational package to inject the external current **j** ∝e^i**kr**−*iωt*^ into the periodic structure and compute the corresponding microscopic electric field **E**_PC_(**r**) generated by this current. In turn, we average the microscopic fields over the unit cell to obtain the macroscopic polarization current and electric field:

(14) $$P\left(\omega ,k\right)=\frac{1}{4\pi S}\int \left(\varepsilon_{\text{PC}}\left(r\right)-1\right)E_{\text{PC}}\left(r\right){\text{e}}^{-\text{i}kr}d^{2}r,$$

(15) $$E\left(\omega ,k\right)=\frac{1}{4\pi S}\int E_{\text{PC}}\left(r\right){\text{e}}^{-\text{i}kr}d^{2}r,$$

where *S* is the area of the unit cell, *ɛ*_PC_(**r**) is the permittivity of the photonic crystal. Finally, we derive the permittivity tensor as a function of both time and spatial frequencies from the corresponding relation:

(16) $$4\pi P\left(\omega ,k\right)=\left[\hat{\varepsilon }\left(\omega ,k\right)-\hat{I}\right]E\left(\omega ,k\right).$$

In all the up-following calculations, we neglect the spatial dispersion effects ${\hat{\varepsilon }}_{\text{MM}}\left(\omega \right)=\hat{\varepsilon }\left(\omega ,k=0\right)$ . The true dependence of the different components of the tensor on the **k**-vector might be found in the Supplemental Materials.

### 6.2 Experimental setup and sample fabrication

For the experimental observation of DSW-II, we fabricated a slab waveguide by printing an array of polylactide plates with a thickness of *a* = 2 mm and a period of *p* = 4 mm using FDM-based 3D printing (Figure S3). Polylactide plastic is commonly used in this 3D printing method. We covered the slabs with aluminum foils to implement the PEC boundary conditions. The dimensions of the fabricated structures are shown in Figure 5(a). The plates in the metamaterial slabs were oriented at an angle of *φ* = ±45° to achieve the required configuration for the existence of DSW-II. We enclosed the system with 2 cm polylactide walls to make the experiment moisture-proof.

Given the low dielectric permittivity of plastic, the anisotropy contrast of a bare air-plastic metamaterial structure is relatively small. Although the small anisotropy itself does not block the existence of DSW-II, it makes the penetration depths *κ*_1,2_ very large compared to the width of the waveguide (see, for example, Figure 2(c)). Even in the centimeters-sized samples, such an aspect ratio is barely realizable. To achieve more significant localization of DSW-II near the interface, we increase the anisotropy constant by infiltrating the space between the plates with water. To further increase the anisotropy contrast, we lower the effective dielectric permittivity of plastic plates by incorporating air voids (not shown in Figure 6(a) and (b)). Finally, as shown in Figure 5, to obtain the highest possible anisotropy contrast at given dielectric permittivities of plastic plates and water, we choose the filling factor of water in the metamaterial to be equal to 0.5. See Supplemental Materials for the dielectric constant of polylactide and water.

To excite the electric field in the structure, we drill a port through the waveguide wall and insert a coaxial input into it. The port is drilled on the interface between two anisotropic metamaterial slabs at 125 mm from the measurement plane, as shown in Figure 5(a). The coaxial input (rod) was inserted inside the port to a depth of approximately 3.1 cm along the *y*-direction, as presented in Figure S4 in the Supplemental Materials. It was observed that such an insertion depth corresponds to the most efficient excitation of DSW-II. Then, the dipole antenna measured the *S*_21_-parameter using a network analyzer in the measurement plane to investigate the electric field distribution.

## Supplementary Material

Supplementary Material Details
