# Supplementary material for: Dyakonov surface waves in dielectric crystals with negative anisotropy
Source: Nanophotonics. 2024 May 9;13(16):3005–15. doi: 10.1515/nanoph-2024-0161 (PMC11501948; doi:10.1515/nanoph-2024-0161)
Supplement: Supplementary file 1 — Supplementary Material Details [file j_nanoph-2024-0161_suppl_001.pdf]

Supplemental Materials to the article:

Dyakonov surface waves in dielectric crystals with negative anisotropy

D. A. Chermoshentsev[1,2,3], E. V. Anikin[1], I. M. Fradkin[1,3], Mikhail S. Sidorenko[4], Aleksandra A. Dudnikova[4], Aleksandr S. Kalganov[4], Mikhail F. Limonov[4,5], N. A. Gippius[1], and S. A. Dyakov[1]

[1]Skolkovo Institute of Science and Technology, Moscow 143025, Russia
[2]Russian Quantum Center, Moscow 143025, Russia
[3]Moscow Institute of Physics and Technology, Dolgoprudny 141700, Russia
[4]ITMO University, 197101 St. Petersburg, Russia
[5]Ioffe Institute, St.Petersburg 194021, Russia

July 2023

1 Waveguide modes of an anisotropic planar waveguide with metallic walls

In this section, we will consider the waveguide modes of a dielectric waveguide $-d/2 < y < d/2$ with permittivity tensor $\hat{\varepsilon}_0 = \mathrm{diag}(\varepsilon_1, \varepsilon_1, \varepsilon_2)$ and metallic walls (PEC boundary condition). The field distributions and dispersion relations for these modes are required for finding the parameters of the effective two–mode theory, in particular, the inverse second derivatives $m_{\mathrm{TE}}^{-1} = m_1^{-1} = \frac{\partial^2}{\partial k_z^2}(\omega_{k_x,k_z}^{\mathrm{TE}})^2\Big|_{k_z=0}$, $m_{\mathrm{TM}}^{-1} = m_2^{-1} = \frac{\partial^2}{\partial k_z^2}(\omega_{k_x,k_z}^{\mathrm{TM}})^2\Big|_{k_z=0}$ and the matrix element

$$\sigma\delta\varepsilon = \int_{-d/2}^{d/2} dy \left(\partial_{k_z}\vec{E}_{k_x,k_z}^{\mathrm{TE}}(y)\delta\hat{\varepsilon}\vec{E}_{k_x k_z'}^{\mathrm{TM}}(y)\right)\Bigg|_{k_z,k_z'=0}, \tag{S1}$$

here $\delta\hat{\varepsilon}$ is a small perturbation of dielectric tensor $\hat{\varepsilon}_0$:

$$\delta\hat{\varepsilon}(z) = \mathrm{sign}(z)\begin{pmatrix} 0 & \delta\varepsilon & 0 \\ \delta\varepsilon & 0 & 0 \\ 0 & 0 & 0 \end{pmatrix}. \tag{S2}$$

The field inside the waveguide is a sum of ordinary and extraordinary waves with y–components

$\pm k_y^o$ and $\pm k_y^e$, where

$$
\begin{aligned}
(k_y^o)^2 &= \frac{\omega^2\varepsilon_1}{c^2} - k_x^2 - k_z^2, \\
(k_y^e)^2 &= \frac{\omega^2\varepsilon_2}{c^2} - k_x^2 - \frac{\varepsilon_2}{\varepsilon_1}k_z^2.
\end{aligned}
\tag{S3}
$$

As the considered waveguide is symmetric with respect to the plane x, z, one can search separately for symmetric and antisymmetric modes. The lowest TE mode is symmetric, and the lowest TM mode is antisymmetric. For symmetric case, the electric field distribution reads

$$
\vec{E} = \frac{A_o c}{\omega}\begin{pmatrix} -k_y^o \cos k_y^o y \\ ik_x \sin k_y^o y \\ 0 \end{pmatrix} + \frac{A_e c^2}{\omega^2}\begin{pmatrix} \varepsilon_2 k_x k_z \cos k_y^e y \\ i\varepsilon_2 k_y^e k_z \sin k_y^e y \\ -\varepsilon_1(k_x^2 + (k_y^e)^2)\cos k_y^e y \end{pmatrix}. \tag{S4}
$$

The PEC boundary condition requires that the electric field components parallel to the waveguide walls are equal to zero. As a result, for the TE-modes, this condition can be satisfied if $A_o = 0$ and, finally, the TE-modes are described as follows:.

$$
\vec{E}^{\text{TE}} = \frac{A_e c^2}{\omega^2}\begin{pmatrix} \varepsilon_2 k_x k_z \cos k_y^e y \\ i\varepsilon_2 k_y^e k_z \sin k_y^e y \\ -\varepsilon_1(k_x^2 + (k_y^e)^2)\cos k_y^e y \end{pmatrix}. \tag{S5}
$$

The dispersion equation of the TE-mode is described by (S6):

$$
(\omega_{k_x,k_z}^{\text{TE}})^2 = c^2((k_y^e)^2 + k_x^2 + \frac{\varepsilon_2}{\varepsilon_1}k_z^2)/\varepsilon_2. \tag{S6}
$$

Finally, m_1^{-1} can be obtained:

$$
m_1^{-1} = \frac{\partial^2}{\partial k_z^2}(\omega_{k_x,k_z}^{\text{TE}})^2\bigg|_{k_z=0} = 2c^2/\varepsilon_1. \tag{S7}
$$

The presence of waveguide walls leads to quantization of the wave vector component $k_y^e = \frac{n\pi}{d}$ perpendicular to them, here n is a waveguide mode order. As the lowest TE-mode in PEC waveguide has $n = 1$, it means that for this waveguide mode $k_y^e = \pi/d$ and the solution for the TE-mode transforms into the following form:

$$
\vec{E}^{\text{TE}} = \frac{A_e c^2}{\omega^2}\begin{pmatrix} \varepsilon_2 k_x k_z \cos \frac{\pi}{d} y \\ i\varepsilon_2 \frac{\pi}{d} k_z \sin \frac{\pi}{d} y \\ -\varepsilon_1(k_x^2 + (\frac{\pi}{d})^2)\cos \frac{\pi}{d} y \end{pmatrix}. \tag{S8}
$$

The modes should be normalized by a condition:

$$
\int (\vec{\mathcal{E}}_{k_x,k_z}^{n}, \hat{\varepsilon}_0 \vec{\mathcal{E}}_{k_x,k_z'}^{n'})\, dydz = 2\pi\delta(k_z - k_z')\delta_{nn'}. \tag{S9}
$$

The normalization give us the coefficient $A_e = \sqrt{\frac{2}{d}}\frac{1}{\varepsilon_2^{3/2}\varepsilon_1}$ and the resulted field (S10):

$$
\vec{E}^{\text{TE}} = \sqrt{\frac{2}{d}}\frac{1}{\varepsilon_2^{3/2}\varepsilon_1}\frac{c^2}{\omega^2}\begin{pmatrix} \varepsilon_2 k_x k_z \cos \frac{\pi}{d} y \\ i\varepsilon_2 \frac{\pi}{d} k_z \sin \frac{\pi}{d} y \\ -\varepsilon_1(k_x^2 + (\frac{\pi}{d})^2)\cos \frac{\pi}{d} y \end{pmatrix}. \tag{S10}
$$

In antisymmetric case, the field distribution inside the waveguide reads

$$\vec{E} = \frac{A_o c}{\omega}\begin{pmatrix} -ik_y^o \sin k_y^o y \\ k_x \cos k_y^o y \\ 0 \end{pmatrix} + \frac{A_e c^2}{\omega^2}\begin{pmatrix} i\varepsilon_2 k_x k_z \sin k_y^e y \\ \varepsilon_2 k_y^e k_z \cos k_y^e y \\ -i\varepsilon_1 (k_x^2 + (k_y^e)^2) \sin k_y^e y \end{pmatrix}. \tag{S11}$$

Finally, from PEC boundary condition for the TM-modes we have that $A_e = 0$ and the field distribution has the following form:

$$\vec{E}^{\text{TM}} = \frac{A_o c}{\omega}\begin{pmatrix} -ik_y^o \sin k_y^o y \\ k_x \cos k_y^o y \\ 0 \end{pmatrix}. \tag{S12}$$

As we want to work with zero-order TM-mode with $n = 0$, it leads to the condition $k_y^o = 0$ and as a result:

$$\vec{E}^{\text{TM}} = \frac{A_o c}{\omega}\begin{pmatrix} 0 \\ k_x \\ 0 \end{pmatrix}. \tag{S13}$$

The dispersion equation of such mode is described by:

$$(\omega_{k_x,k_z}^{\text{TM}})^2 = \frac{c^2((k_y^o)^2 + k_x^2 + k_z^2)}{\varepsilon_1}. \tag{S14}$$

And, analogically to TE-mode the coefficient $m_2^{-1} = 2c^2/\varepsilon_1$.

After the normalization (S9), the TM-mode gets the following form:

$$\vec{E}^{\text{TM}} = \frac{1}{\varepsilon_1 \sqrt{d}} \frac{c}{\omega}\begin{pmatrix} 0 \\ k_x \\ 0 \end{pmatrix}. \tag{S15}$$

The calculation of the TE-TM modes matrix element σ by (S1) gives us:

$$\sigma = \frac{2\sqrt{2\varepsilon_2}}{\varepsilon_1^{3/2}\pi} \frac{k_x}{k_x^2 + (\frac{\pi}{d})^2}. \tag{S16}$$

The intersection point of TM and TE modes can be obtained as follow:

$$k_x d = \pi\sqrt{\frac{\varepsilon_1}{\varepsilon_2 - \varepsilon_1}}. \tag{S17}$$

2 Low anisotropy approximation

As was shown in Ref. [1] the field distribution of DSWM-II could be obtained as an expansion over the lowest TM and TE modes:

$$\vec{E}(y,z) = \int \frac{dk_z}{2\pi}\left[\alpha(k_z)\vec{\mathcal{E}}_{k_x,k_z}^{\text{TE}} + \beta(k_z)\vec{\mathcal{E}}_{k_x,k_z}^{\text{TM}}\right], \tag{S18}$$

where $\vec{\mathcal{E}}^{\text{TE(TM)}}_{k_x,k_z}(y,z) = \vec{E}^{\text{TE(TM)}}_{k_x,k_z}(y)e^{ik_z z}$, and $\alpha(k_z)$ and $\beta(k_z)$ are slowly varying envelopes whose Fourier images $\alpha(z)$ and $\beta(z)$ could be obtained from the following system of equations:

$$\begin{pmatrix} \gamma^{\text{TE}}_{k_x,0} - \frac{1}{2m_1}\frac{\partial^2}{\partial z^2} & i\omega^2\sigma\delta\varepsilon\partial_z\,\text{sign}(z) \\ i\omega^2\sigma\delta\varepsilon\,\text{sign}(z)\partial_z & \gamma^{\text{TM}}_{k_x,0} - \frac{1}{2m_2}\frac{\partial^2}{\partial z^2} \end{pmatrix} \begin{pmatrix} \alpha(z) \\ \beta(z) \end{pmatrix} = 0, \tag{S19}$$

where $\gamma^{\text{TE(TM)}}_{k_x,k_z} = \left(\omega^{\text{TE(TM)}}_{k_x,k_z}\right)^2 - \omega^2$.

Let us note that the envelopes should also obey the following boundary condition:

$$\begin{cases} \partial_z\alpha(+0) - \partial_z\alpha(-0) = 4im_1\omega^2\sigma\delta\varepsilon\beta(0), \\ \partial_z\beta(z) \textit{ is continuous at } z = 0. \end{cases} \tag{S20}$$

Finally, the exponentially decaying solution of Eq. S19 which satisfies the boundary conditions (S20) is described as follows:

$$\begin{gathered} \alpha(z) = \frac{\omega^2\sigma\delta\varepsilon e^{-\kappa_2|z|}}{\left(\gamma^{\text{TE}}_{k_x,0} - \frac{\kappa_2^2}{2m_1}\right)} - \frac{\omega^2\sigma\delta\varepsilon e^{-\kappa_1|z|}}{\left(\gamma^{\text{TE}}_{k_x,0} - \frac{\kappa_1^2}{2m_1}\right)}, \\ \beta(z) = -i\left(\frac{e^{-\kappa_2|z|}}{\kappa_2} - \frac{e^{-\kappa_1|z|}}{\kappa_1}\right), \end{gathered} \tag{S21}$$

here parameters $\kappa_{1,2}$ are roots with positive real part of the characteristic equation:

$$\left(\gamma^{\text{TE}}_{k_x,0} - \frac{\kappa^2}{2m_1}\right)\left(\gamma^{\text{TM}}_{k_x,0} - \frac{\kappa^2}{2m_2}\right) + \omega^4\sigma^2\delta\varepsilon^2\kappa^2 = 0. \tag{S22}$$

After substitution (S10), (S13) and (S21) into the Eq. S19 the explicit solution of DWM gets the following view:

$$\vec{E}^{\text{DWM}}(y,z) = \frac{c}{\omega\varepsilon_1\sqrt{d}}\left[\frac{\sqrt{2}}{\varepsilon_2^{3/2}}\frac{c}{\omega}\begin{pmatrix} -i\partial_z\alpha(z) \\ -i\partial_z\alpha(z) \\ \alpha(z) \end{pmatrix}\begin{pmatrix} \varepsilon_2 k_x\cos\frac{\pi}{d}y \\ i\varepsilon_2\frac{\pi}{d}\sin\frac{\pi}{d}y \\ -\varepsilon_1(k_x^2 + (\frac{\pi}{d})^2)\cos\frac{\pi}{d}y \end{pmatrix} + \beta(z)\begin{pmatrix} 0 \\ k_x \\ 0 \end{pmatrix}\right]. \tag{S23}$$

The dispersion equation of DSWM-II could be obtained by substitution the expressions for slowly varying envelopes (S21) into the boundary condition (S20):

$$\sqrt{m_1 m_2\gamma^{\text{TE}}_{k_x,0}\gamma^{\text{TM}}_{k_x,0}} = 2m_1m_2(\omega^2\sigma\delta\varepsilon)^2 - m_2\gamma^{\text{TM}}_{k_x,0}. \tag{S24}$$

3 Influence of high order waveguide modes

Since the developed model considers only the two lowest waveguide modes as a basis of expansion, the spacing between the two first modes and higher-order modes becomes crucial. If this spacing is not large enough S1, high order modes can influence on the structure of DSWM-II. As a result, the DSWM-II field calculated by Eq. S23 and the exact solution of Maxwell's equations can have some differences (see Fig.2b in the main text). The spacing between modes can be increased by

considering waveguides with larger anisotropy (Fig. S1a,b), and the influence of high order modes can be reduced. The field distributions of DSWM-II for the waveguide with $\varepsilon_1 = 9$, $\varepsilon_2 = 12.75$ and $\delta\varepsilon = 0.375$ are presented in Fig. S2. In this case, the spacing between the first two waveguide modes and high order modes is large. Therefore the results of theoretical calculations and numerical simulation provided by Comsol Multiphysics are in good agreement. Let us note that the increase of ε_2 could be realized using biaxial anisotropic crystals instead of uniaxial as materials formed the waveguide.

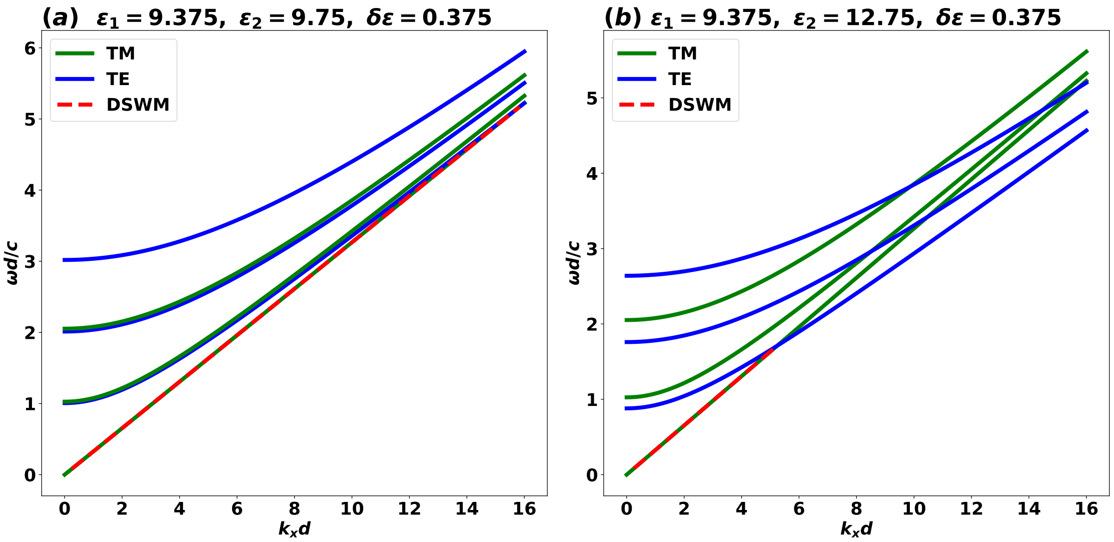

Figure S1: The dispersions of the DWM (red dashed line) and the TE and TM waveguide modes of an anisotropic waveguide (blue and green solid lines) calculated for $\lambda = 1550\,\text{nm}$, $d = 420\,\text{nm}$. and dielectric permittivities: a) $\varepsilon_1 = 9.375$, $\varepsilon_2 = 9.75$, $\delta\varepsilon = 0.375$; b) $\varepsilon_1 = 9.375$, $\varepsilon_2 = 12.75$, $\delta\varepsilon = 0.375$.

4 Experimental investigation of Dyakonov Fabry-Pérot resonance

The periodic array of polylactide samples with a dielectric permittivity of $\varepsilon_{PLA} = 1.7 + i10^{-5}$ was printed, and water filled the spaces between the plates to investigate the Dyakonov Fabry-Pérot resonances. The measured real and imaginary parts of the dielectric permittivity of the water are presented in Fig. S3, as well as the theoretically calculated values using the approach presented in Ref. [2]. The measurement of the imaginary part of the dielectric function for frequencies less than 1 GHz is complicated due to the lack of sensitivity of the receiver. Nevertheless, we find good agreement between the experimental and theoretical results for the higher frequencies. The geometrical parameters of the structure are described in the manuscript's main text. The excitation of the eigenmodes is realized by coaxial input in the scheme shown in Fig. S4. The length of the coaxial port inside the waveguide is chosen due to the maximum level of the S21 coefficient.

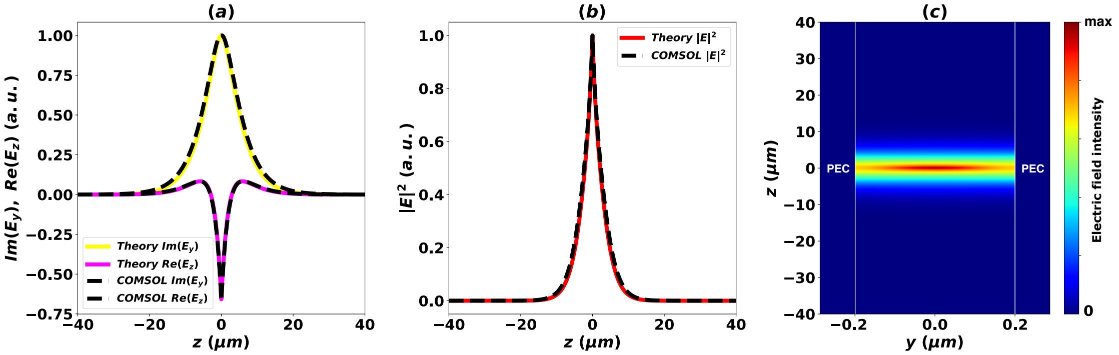

Figure S2: (a) Theoretically calculated fields $E_y(0,z)$ and $E_z(0,z)$ (yellow and magenta lines) shown together with COMSOL simulation results (black dashed lines). (b) Theoretically calculated electric field intensity $|E|^2(0,z)$ (red solid line) shown together with COMSOL simulation results (black dashed line). (c) Theoretically calculated electric field intensity in DWM. Please note that we use different scales for the y- and z-axis. Calculations are made for $\lambda = 1550\,\mathrm{nm}$, $k_x d = 5.2135$ and $d = 420\,\mathrm{nm}$, $\varepsilon_1 = 9.375$, $\varepsilon_2 = 12.75$, $\delta\varepsilon = 0.375$.

In the considered 3D structure, the field distribution of DSWM is asymmetric relative to the interface. The reason for such an anisotropy is that the Dyakonov Fabry-Pérot resonance is a superposition of two Dyakonov surface waves propagated in opposite directions. The electric energy $|E|^2$ distribution of DSWM propagated in the forward direction is shown in Fig. S5a. The electric energy distribution of the superposition of two DSWM propagated in forward and backward directions with the phase shift $\theta = \pi/2$, resulting from the normal reflection presented in Fig. S5b. The electric energy distribution of Dyakonov Fabry-Pérot resonance obtained by direct 3D simulation is presented in Fig. S5c.

5 Polarization of the Dyakonov Surface Fabry-Pérot resonances

To investigate the Dyakonov Surface Fabry-Pérot resonance's polarization, we also developed the model in Comsol Multiphysics, reproduced in the experimental setup. We considered a model the same size as the experimental sample and excited the field with frequency f by the coaxial port using a Frequency Domain solver. The scheme of the model in Comsol was presented in Fig. S6a, and the example of the calculated field is presented in Fig. S6b.

To compare the polarization of the mode, we used the in-phase representation of the DSWM field for experimentally measured field distribution (Fig. S7a), for the field calculated by eigenmode analysis (Fig. S7b) and for the numerically calculated field using the excitation through the coaxial input (Fig. S7c). One can see that the experimental results have an additional elliptically part of the polarization, resulting from the complexes of the excitation and measurement systems.

6 Derivation of effective dielectric tensor of anisotropic metamaterial

The real and imaginary components of dielectric permittivity tensor calculated by the approach presented in Section Methods of the manuscript are shown in Fig. S8 and Fig. S9 correspondingly.

7 Measurement of water dielectric permittivity

Before conducting the research, the dielectric constant of distilled water used in the experimental setup was measured. SPEAG Dielectric Assessment Kit [1] and VNA Agilent Technologies PNA E8362C were used as measurement tools. Before carrying out measurements, it is necessary to calibrate the device. Chemically pure methyl alcohol was used as a calibration measure. The probe used is DAK 3.5, IF Bandwidth 100 Hz, output power 0 dBm, time averaging 3. As stated above, chemically pure methyl alcohol was used as a load, air was used as an idle circuit, and a copper strip was used as a short circuit.

After calibration, the probe is wiped and then immersed in water at a temperature of 20 degrees Celsius. Using software, the dielectric constant of water is determined (81).

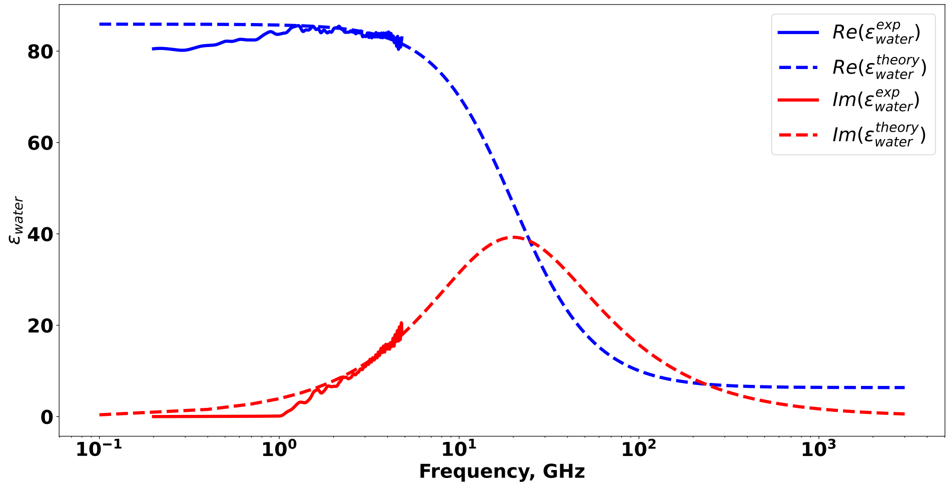

Figure S3: Measured frequency dependence of the real and imaginary parts of the dielectric permittivity of water (solid) and the numerically calculated permittivity by Ref. [2] (dashed).

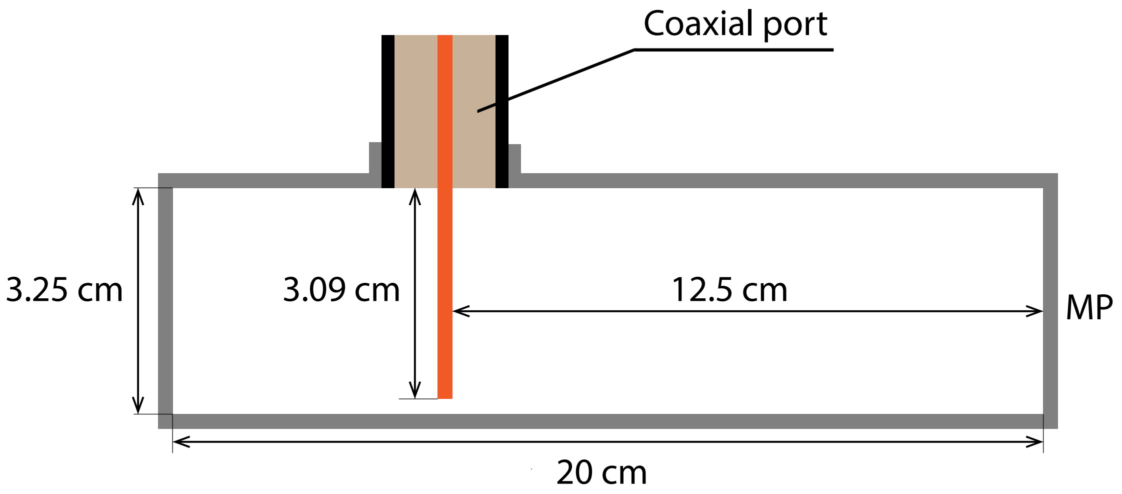

Figure S4: The excitation scheme of DSW-II. The field with frequency f is excited through the coaxial port and measured by the dipole antenna along the measurement plane (MP).

8 Dyakonov surface waves for different angles between optical axes

We analyzed the possibility of the existence of surface states at various angles between the optical axes of the materials forming the interface. We discovered that at a fixed wavelength, λ, decreasing the angle causes the DSW-II to transition into a TM waveguide mode, while increasing the angle shifts it into a TE mode, as illustrated in the Fig. S10 and Fig. S11. Subsequently, we investigated how the angle between the optical axes affects the generation of surface states in an experimental sample. We found that decreasing the angle decreases the frequency of the surface resonance, while increasing it causes the frequency to increase. At angles of 60° and 110°, the mode becomes significantly delocalized, gradually transitioning into a bulk mode as shown in Fig. S12.

References

[1] E. V. Anikin, D. A. Chermoshentsev, S. A. Dyakov, and N. A. Gippius, "Dyakonov-like waveguide modes in an interfacial strip waveguide," *Physical Review B*, vol. 102, p. 161113(R), oct 2020.

[2] R. E. Jacobsen, S. Arslanagić, and A. V. Lavrinenko, "Water-based devices for advanced control of electromagnetic waves," *Applied Physics Reviews*, vol. 8, dec 2021.

[3] S. A. Dyakov, V. A. Semenenko, N. A. Gippius, and S. G. Tikhodeev, "Magnetic field free circularly polarized thermal emission from a chiral metasurface," *Physical Review B*, vol. 98, p. 235416, dec 2018.

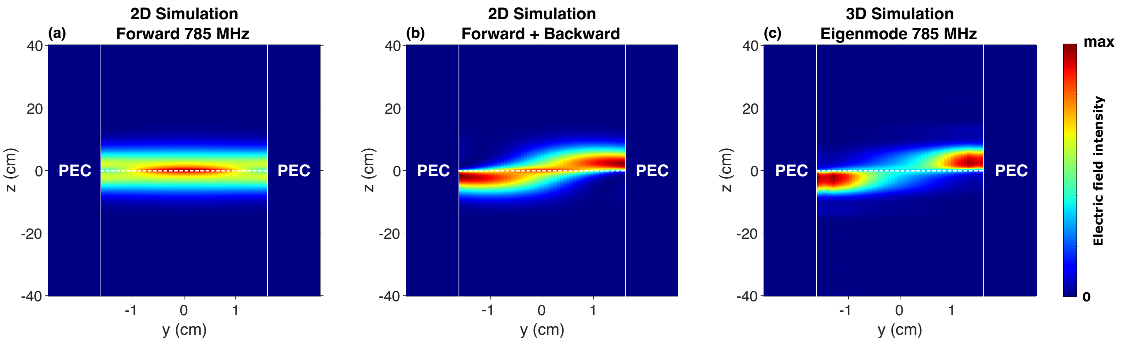

Figure S5: (a) Calculated field distribution of DSWM-II type propagating along the X-axis in a forward direction in an interfacial strip waveguide with cross-section and material parameters corresponding to the experimental sample; (b) The sum of the calculated field distribution of DSWMs-II propagating along the X-axis in forward and backward directions. The phase of the backward wave is rotated on $\varphi = \pi/2$; (c) 3D Simulation of Dyakonov surface Fabry-Pérot resonance field distribution in the experimental sample.

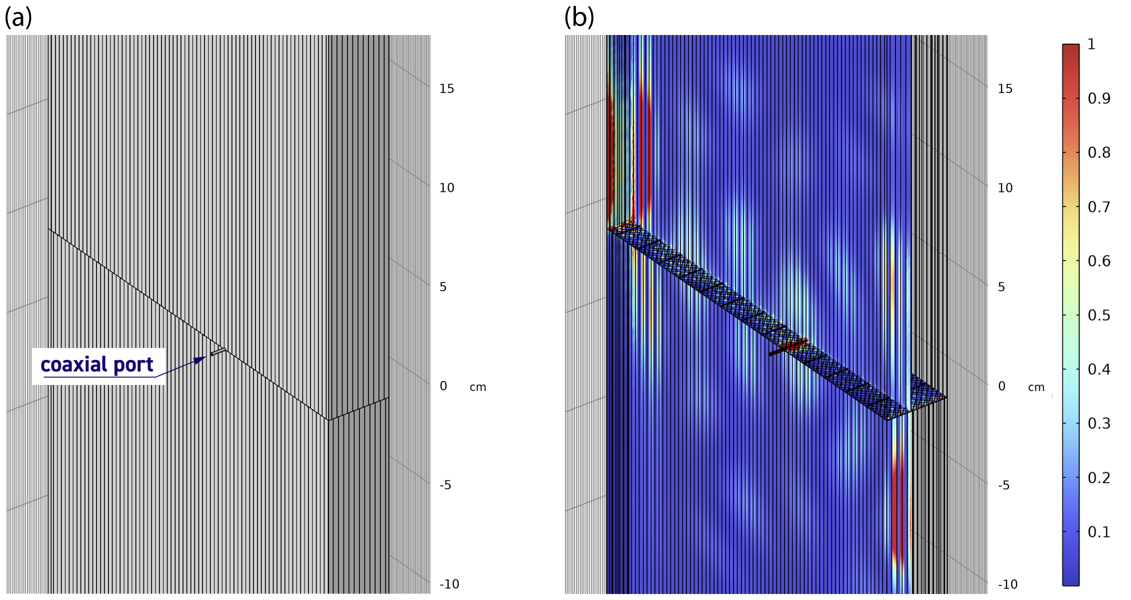

Figure S6: (a) The model in Comsol Multiphysics reproduced the experimental setup of the Dyakonov Fabry-Pérot Resonances measurements; (b) The example of the electric field normalized intensity distribution for mode excited with frequency $f = 790$ MHz.

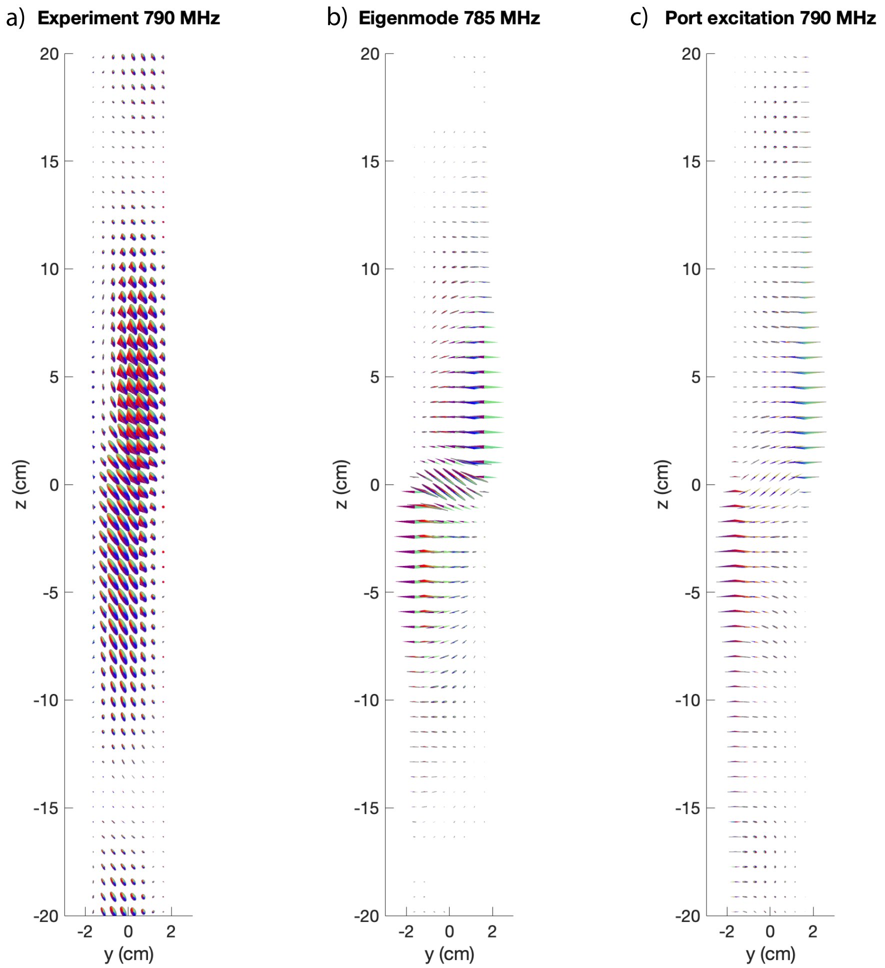

Figure S7: The in-phase representation of the electric field of a) experimentally measured Dyakonov Fabry-Pérot resonance; b) numerically calculated Dyakonov Fabry-Pérot resonance; c) numerically excited Dyakonov Fabry-Pérot resonance by coaxial port. In the phase representation, we intend to show the field orientations during the entire period of electromagnetic oscillations. In such representation, i) the cone base lies in the polarization plane where the field oscillates; ii) in the most general case, the cone base is an ellipse circumscribed by the field vector during one oscillating period. In the case of linear polarization, the ellipse degenerates into a straight line, while in the case of purely circular polarization, the ellipse degenerates into a circle; iii) the cone height is equal to the product of the electric field amplitude, and the circular polarization degree; iv) the direction of the cone follows right screw rule; v) the color scale represents the phase of electromagnetic oscillations. See Ref. [3] for the details.

.

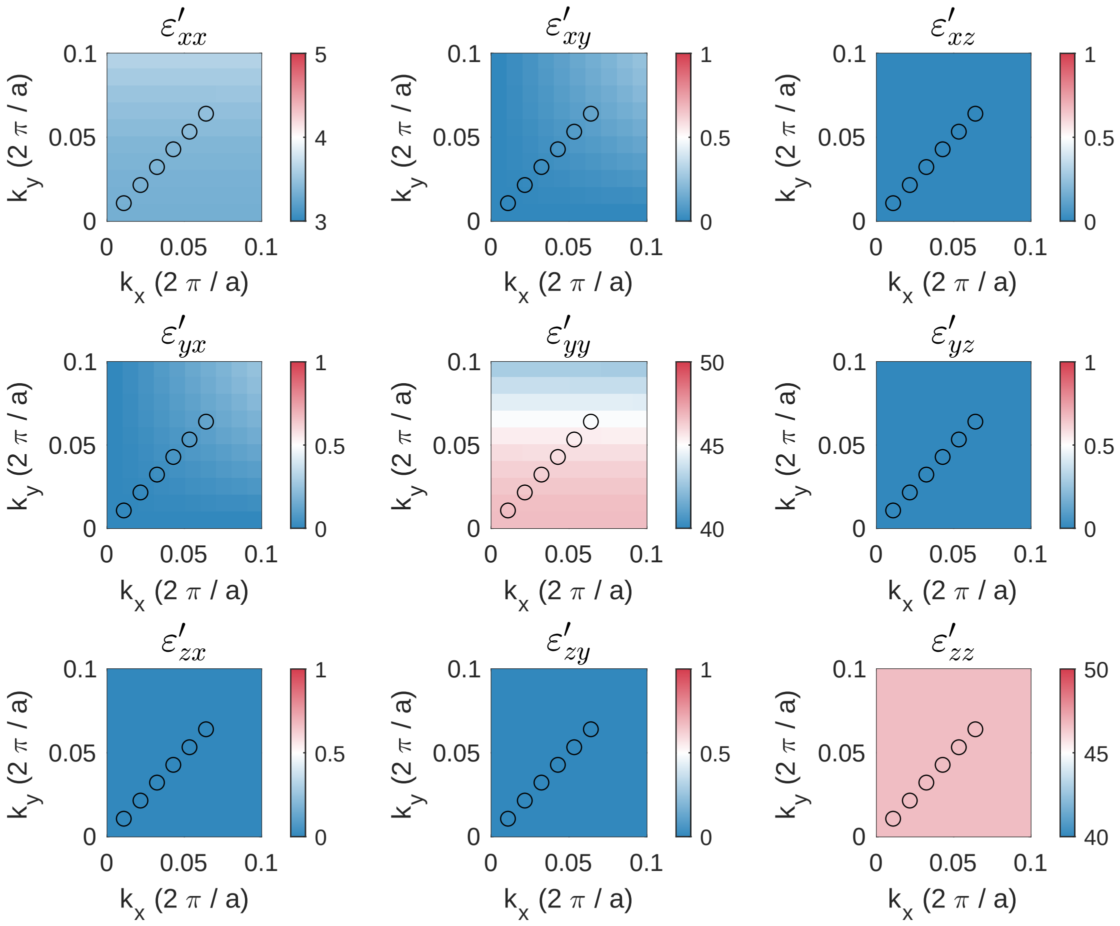

Figure S8: The real parts of dielectric permittivity tensor components calculated by the effective media theory have considered spatial dispersion. The circles highlighted the Dyakonov surface Fabry-Pérot resonances in the experimental sample.

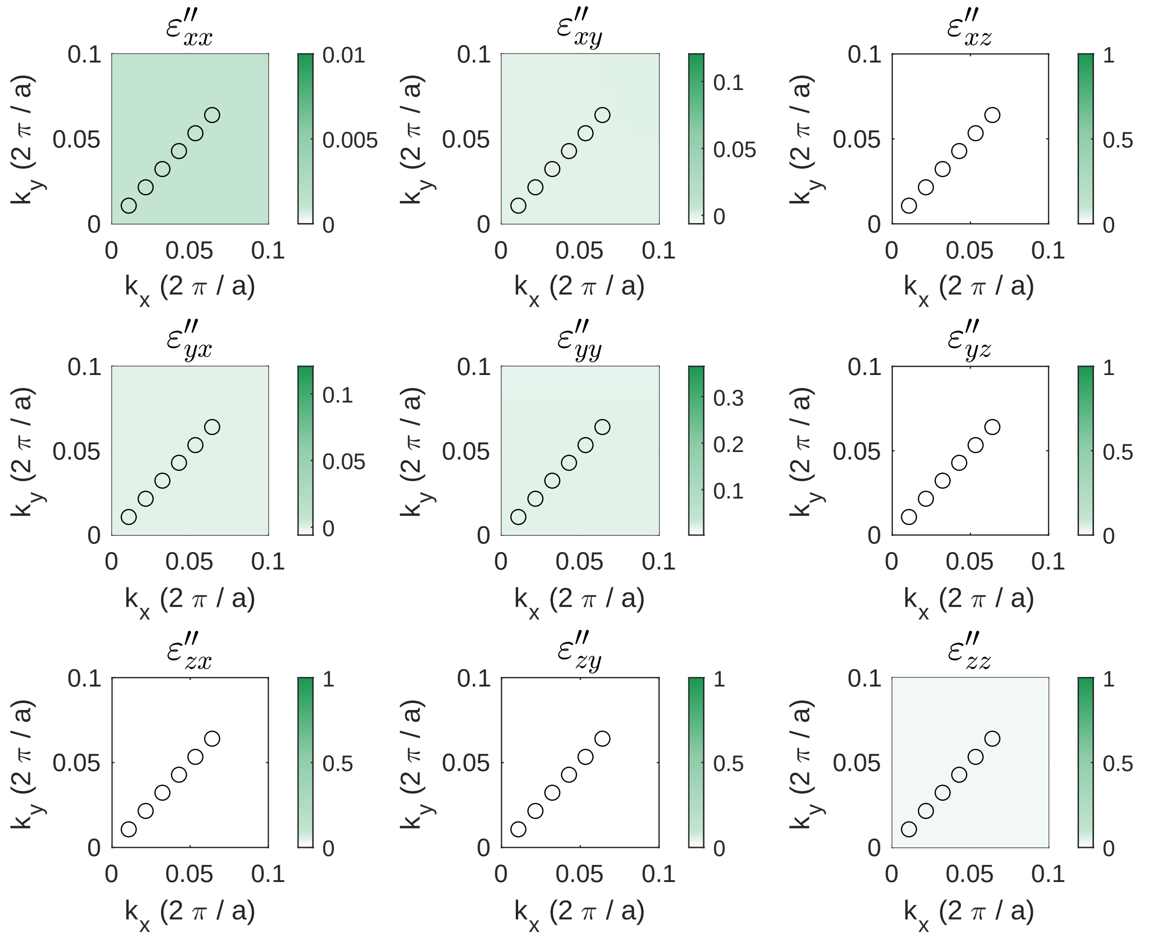

Figure S9: The imaginary parts of dielectric permittivity tensor components calculated by the effective media theory have considered spatial dispersion. The circles highlighted the Dyakonov surface Fabry-Pérot resonances in the experimental sample.

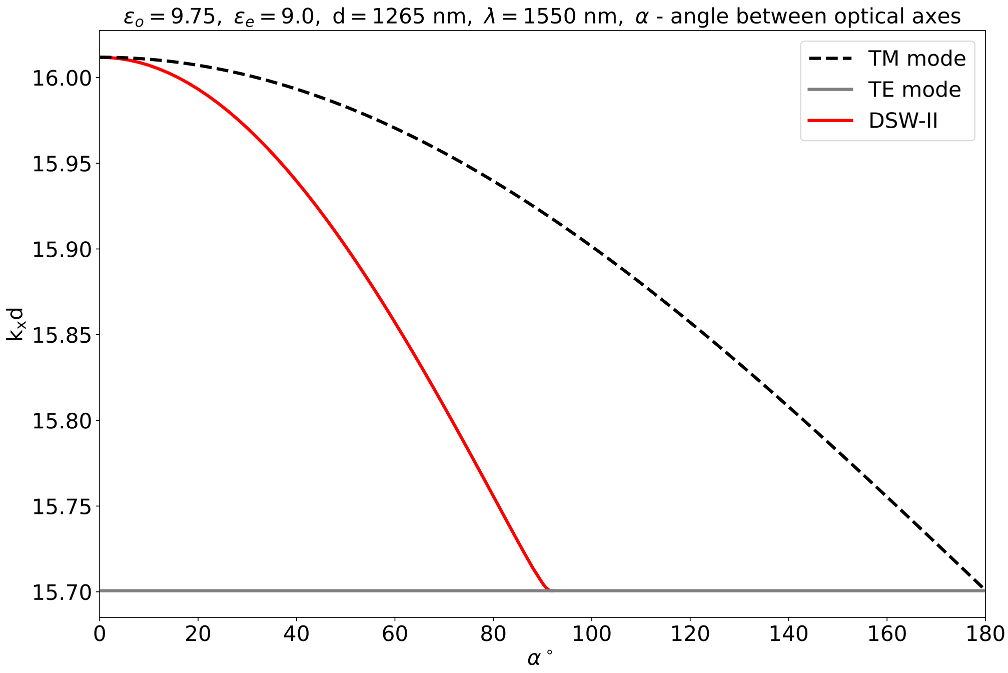

Figure S10: The dependence of the DSW-II propagation constant on the angle between the optical axes α. Calculations are made for $\lambda = 1550\,\mathrm{nm}$, and $d = 1265\,\mathrm{nm}$. Dielectric permittivities are $\varepsilon_o = 9.75$, $\varepsilon_e = 9.0$.

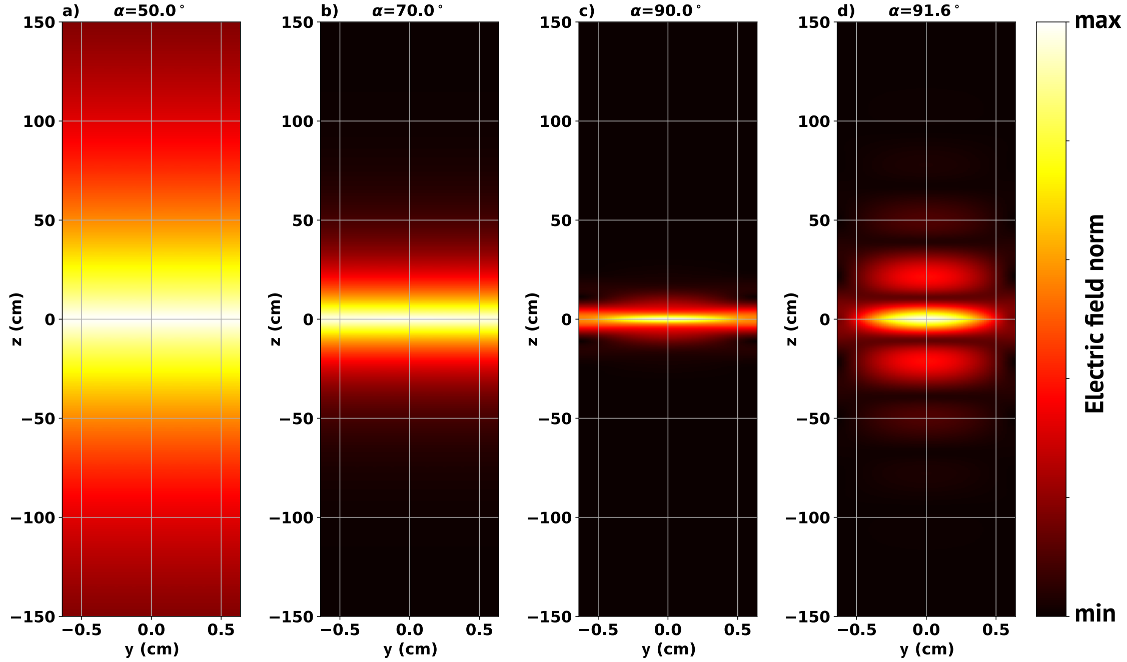

Figure S11: (a)-(d) Numerically calculated electric energy density in DSW-II for different angles between optical axes α. Please note that the limits of y- and z-axes are different. Calculations are made for $\lambda = 1550\,\text{nm}$, and $d = 1265\,\text{nm}$. Dielectric permittivities for all panels are $\varepsilon_o = 9.75$, $\varepsilon_e = 9.0$.

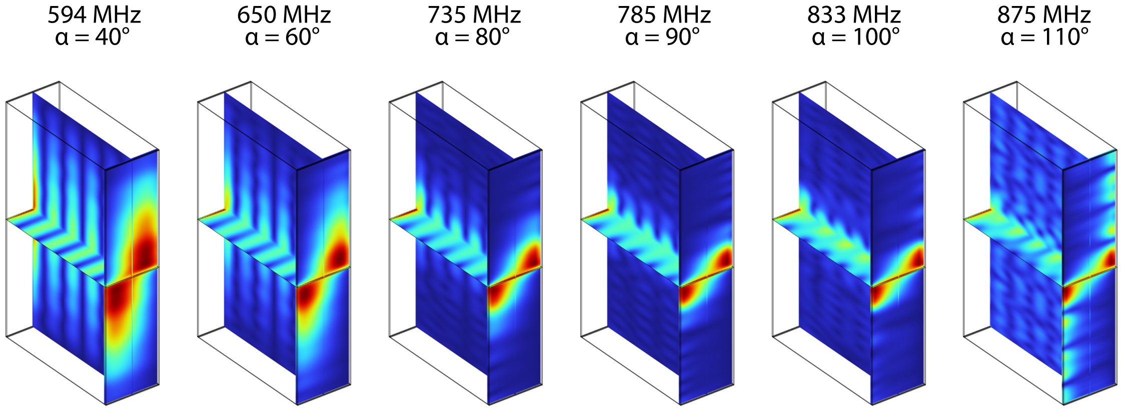

Figure S12: (a)-(d) Numerically calculated electric energy density in Fabry-Pérot-like DSWs-II calculated for $N = 5$ for different angles between optical axes α. Please note that the limits of y- and z-axes are different. The calculation are performed for the parameters of experimental sample.
